# Identification of ester-linked ubiquitylation sites during TLR7 signalling increases the number of inter-ubiquitin linkages from 8 to 12

**DOI:** 10.1042/BCJ20220510

**Published:** 2022-12-07

**Authors:** Elisha H. McCrory, Vyacheslav Akimov, Philip Cohen, Blagoy Blagoev

**Affiliations:** 1 MRC Protein Phosphorylation and Ubiquitylation Unit, University of Dundee, Dundee, Scotland, U.K.; 2 Department of Biochemistry and Molecular Biology, University of Southern Denmark, Odense, Denmark

**Keywords:** HOIL-1, innate immunity, Toll-like receptors, ubiquitin ligases, ubiquitins

## Abstract

The E3 ligase HOIL-1 forms ester bonds *in vitro* between ubiquitin and serine/threonine residues in proteins. Here, we exploit UbiSite technology to identify serine and threonine residues undergoing HOIL-1 catalysed ubiquitylation in macrophages stimulated with R848, an activator of the TLR7/8 heterodimer. We identify Thr12, Thr14, Ser20 and Thr22 of ubiquitin as amino acid residues forming ester bonds with the C-terminal carboxylate of another ubiquitin molecule. This increases from 8 to 12 the number of ubiquitin linkage types that are formed in cells. We also identify Ser175 of IRAK4, Ser136, Thr163 and Ser168 of IRAK2 and Thr141 of MyD88 as further sites of HOIL-1-catalysed ubiquitylation together with lysine residues in these proteins that also undergo R848-dependent ubiquitylation. These findings establish that the ubiquitin chains attached to components of myddosomes are initiated by both ester and isopeptide bonds. Ester bond formation takes place within the proline, serine, threonine-rich (PST) domains of IRAK2 and IRAK4 and the intermediate domain of MyD88. The ubiquitin molecules attached to Lys162, Thr163 and Ser168 of IRAK2 are attached to different IRAK2 molecules.

## Introduction

The linear ubiquitin assembly complex (LUBAC) contains two different E3 ubiquitin ligases, HOIL-1 (haem-oxidised IRP2 ubiquitin ligase-1), also called RBCK1 (RING-B-box-coiled-coil protein interacting with PKC 1), and HOIP (HOIL-1-interacting protein). HOIP catalyses the formation of Met1-linked ubiquitin (M1-Ub, also called linear ubiquitin) in which the α-amino group of the N-terminal methionine residue of ubiquitin forms a peptide bond with the C-terminal glycine residue of another ubiquitin molecule [1]. In contrast, HOIL-1 is one of the few E3 ligases so far identified that forms ester bonds between the C-terminal carboxylate of ubiquitin and serine and threonine residues in proteins [2,3], the great majority of E3 ligases forming isopeptide bonds between the C-terminal glycine of ubiquitin and ε-amino side chains of lysine residues, including the seven lysine residues in ubiquitin [4].

One well-established physiological role of LUBAC is to regulate innate immune signalling pathways in which M1-Ub oligomers formed by HOIP interact with NEMO (NF-κB essential modulator) [5,6], a component of the canonical IκB kinase (IKK) complex. This induces a conformational change [7] that facilitates IKK activation by the TAK1 kinase complex [8,9], leading to activation of the transcription factors NF-κB and IRF5 (interferon regulatory factor 5) [10,11] that are essential for transcription of genes encoding some of the inflammatory mediators needed to mount responses that combat infection by microbial pathogens. The M1-Ub oligomers also interact with A20 [12,13] and ABIN1 (A20-binding inhibitor of NF-κB 1), which function to restrict TAK1 and IKK activation, preventing the overproduction of inflammatory mediators that cause inflammatory and autoimmune diseases [14,15]. M1-Ub formed by HOIP, therefore, has both positive and negative roles in controlling inflammatory mediator production.

The HOIL-1 E3 ligase also appears to have both positive and negative roles in the regulation of immunity. Bone marrow-derived macrophages (BMDM) from mice in which wild-type HOIL-1 is replaced by the E3 ligase-inactive HOIL-1[C458S] mutant produce reduced amounts of the pro-inflammatory cytokines IL-6 and IL-12 in response to TLR (Toll-like receptor)-activating ligands, whereas cytotoxic T cells from the same mice produce enhanced amounts of interferon γ and Granulocyte Macrophage Colony Stimulating Factor in response to IL-18 [16]. HOIL-1-catalysed ester-linked ubiquitylation events can, therefore, decrease or increase inflammatory mediator production, depending on the ligand, signalling pathway and cell type. This is consistent with the observation that HOIL-1-deficient humans display a combination of immunodeficiency and auto-inflammation [17,18]. However, the molecular mechanism by which ester-linked ubiquitylation regulates immunity is unknown.

IL-18, other IL-1 family members and TLRs all initiate intracellular signalling by forming myddosomes [19,20], which are large oligomeric complexes comprising MyD88 (myeloid differentiation primary response gene 88), IRAK4 (interleukin receptor-associated kinase 4) and IRAKs 1 and 2. All four myddosome components undergo polyubiquitylation during IL-1 or TLR signalling, the ubiquitin oligomers attached to these proteins containing both Lys63- ubiquitin (K63-Ub) linkages formed by the E3 ligases TRAF6, Pellino 1 and Pellino 2 [21–23] and M1-Ub linkages formed by HOIP [24]. Some, but not all of these hybrid ubiquitin chains, appear to be initiated by the formation of HOIL-1-catalysed ester bonds, based on their cleavage at mildly alkaline pH by the nucleophile hydroxylamine, and the absence of hydroxylamine-sensitive bonds in macrophages from HOIL-1[C458S] mice [2]. However, to establish whether these inferences are correct requires identification of the specific amino acid residues to which ubiquitin is attached. Here, we have used UbiSite technology to conduct this analysis [25], because it enables sites of ester-linked and isopeptide-linked ubiquitylation to be identified simultaneously in the same experiment. These studies have also revealed that HOIL-1 forms four different ester bonds between two ubiquitin molecules during TLR signalling.

## Results and discussion

### Some of the ubiquitin chains attached to IRAK4 during R848 signalling are cleaved by hydroxylamine

**Figure 1.**
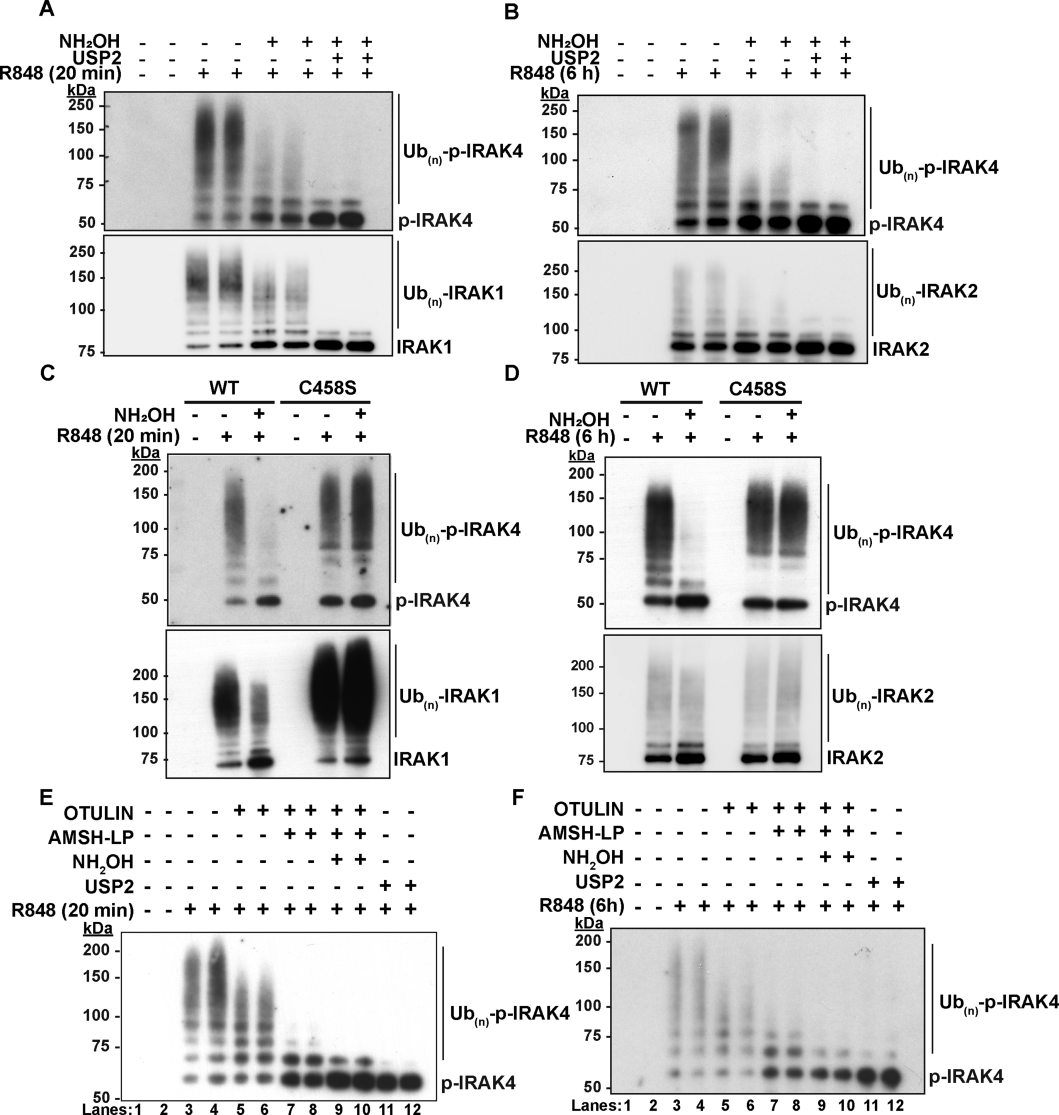
Some of the ubiquitin chains attached to IRAK4 in R848-stimulated macrophages are initiated by ester bond formation catalysed by HOIL-1. (**A**,**B**) RAW264.7 cells were stimulated for 20 min (**A**) or 6 h (**B**) with the TLR7-activating ligand R848 (250 ng/ml) and ubiquitylated proteins were captured from the cell extracts on Halo-NEMO beads. The Halo-NEMO pull downs were incubated for 1 h without (−) or with (+) 0.5 M hydroxylamine at pH 9.0 followed by incubation without (−) or with (+) the broad specificity deubiquitylase USP2 (1 μM). (C,D) The ubiquitylated proteins captured from the extracts of BMDM from wild-type (WT) or HOIL-1[C458S] mice (C458S) were incubated for 1 h without (−) or with (+) 0.5 M hydroxylamine at pH 9.0 and analysed as in (A,B). (E,F) As in (A B) except that, prior to SDS–PAGE and immunoblotting, the Halo-NEMO resin was incubated with 1 µM Otulin (lanes 5 and 6), 1 µM Otulin plus 1 µM AMSH-LP (lanes 7 and 8) and Otulin plus AMSH-LP and then 0.5 M hydroxylamine (lanes 9 and 10), or 1 µM USP2 alone (lanes 11 and 12) (see Methods).

Although IRAK4 undergoes polyubiquitylation during TLR ligation [24], whether the ubiquitin chains attached to this myddosome component are initiated by both isopeptide and ester bonds has not been investigated. Here, we found that, as for IRAK1 and IRAK2 (lower panels of Figure 1A,B), some of the ubiquitylated IRAK4 formed after stimulation of RAW264.7 cells (RAW cells) for 20 min or 6 h with R848 (upper panels of Figure 1A,B) are converted to unmodified IRAK4 by treatment with hydroxylamine. Thus, some of the ubiquitin chains attached to IRAK4 also appear to be initiated by ester bond formation.

To establish whether these putative ester bonds are catalysed by HOIL-1, we repeated the experiments with primary BMDM from mice expressing the E3 ligase-inactive HOIL-1[C458S] mutant. Similar to the RAW cells, the ubiquitylated IRAK4 in primary BMDM from wild-type (WT) mice is partially converted to deubiquitylated IRAK4 by incubation with hydroxylamine. However, the ubiquitylated IRAK4 formed in BMDM from HOIL-1[C458S] mice is resistant to this nucleophile (Figure 1C,D). The results indicated that the formation of hydroxylamine-sensitive bonds in ubiquitylated IRAK4 requires HOIL-1 E3 ligase activity.

Treatment with Otulin, a deubiquitylase (DUB) that hydrolyses M1-Ub linkages specifically [26,27], reduced the size of the ubiquitin chains attached to IRAK4, without converting IRAK4 to either the monoubiquitylated or deubiquitylated species, while treatment with Otulin followed by treatment with AMSH-LP (associated molecule with the SH3-domain of STAM-like protease), a DUB that hydrolyses K63-Ub linkages specifically [28], largely converted ubiquitylated IRAK4 to monoubiquitylated and deubiquitylated species (Figure 1E,F). The observation that AMSH-LP causes partial cleavage of the first ubiquitin attached to some proteins has been noted previously [24]. Taken together, the results indicate that, similar to IRAK1 and IRAK2, the K63/M1-Ub chains attached to IRAK4 are initiated by both ester bonds and isopeptide bonds.

### Identification of serine, threonine and lysine residues in myddosome components that undergo ubiquitylation during TLR7/8 signalling

Ubiquitin comprises 76 amino acid residues and terminates in the sequence Arg-Gly-Gly. To identify sites of ubiquitylation, a protocol has been developed in which ubiquitylated proteins are digested with trypsin to cleave the Arg-Gly bond between amino acid residues 74 and 75 of ubiquitin, generating peptides containing characteristic ‘Gly-Gly' (diGly) signatures that can be enriched by immunoprecipitation with an antibody recognising diGly-isopeptide sequences prior to their identification by mass spectrometry (MS) [29–31]. However, these antibodies do not recognise diGly sequences attached by ester bonds to the hydroxyl side chains of serine or threonine residues, nor do they distinguish between lysine residues forming isopeptide bonds with ubiquitin or with the two ubiquitin-like modifiers (UBLs) ISG15 (Interferon-Stimulated Gene 15) and NEDD8 (neuronal precursor cell-expressed developmentally down-regulated protein 8), both of which also terminate in a diGly sequence following tryptic digestion. To circumvent these problems, we, therefore, performed an initial digestion with LysC proteinase to generate ubiquitylated peptides in which amino acid residues 64–76 of ubiquitin are linked to peptides from the modified proteins. These peptides are enriched by immunoprecipitation with ‘UbiSite', an antibody that recognises amino acids 64–76 of ubiquitin but not the C-terminal peptides from other UBLs [25]. Re-digestion with trypsin, now generates peptides in which the diGly signature from ubiquitin is linked to Ser/Thr, Lys or Met-1 residues, which are identified by MS. A schematic summarising the methodology used is shown in Supplementary Figure S1.

**Figure 2.**
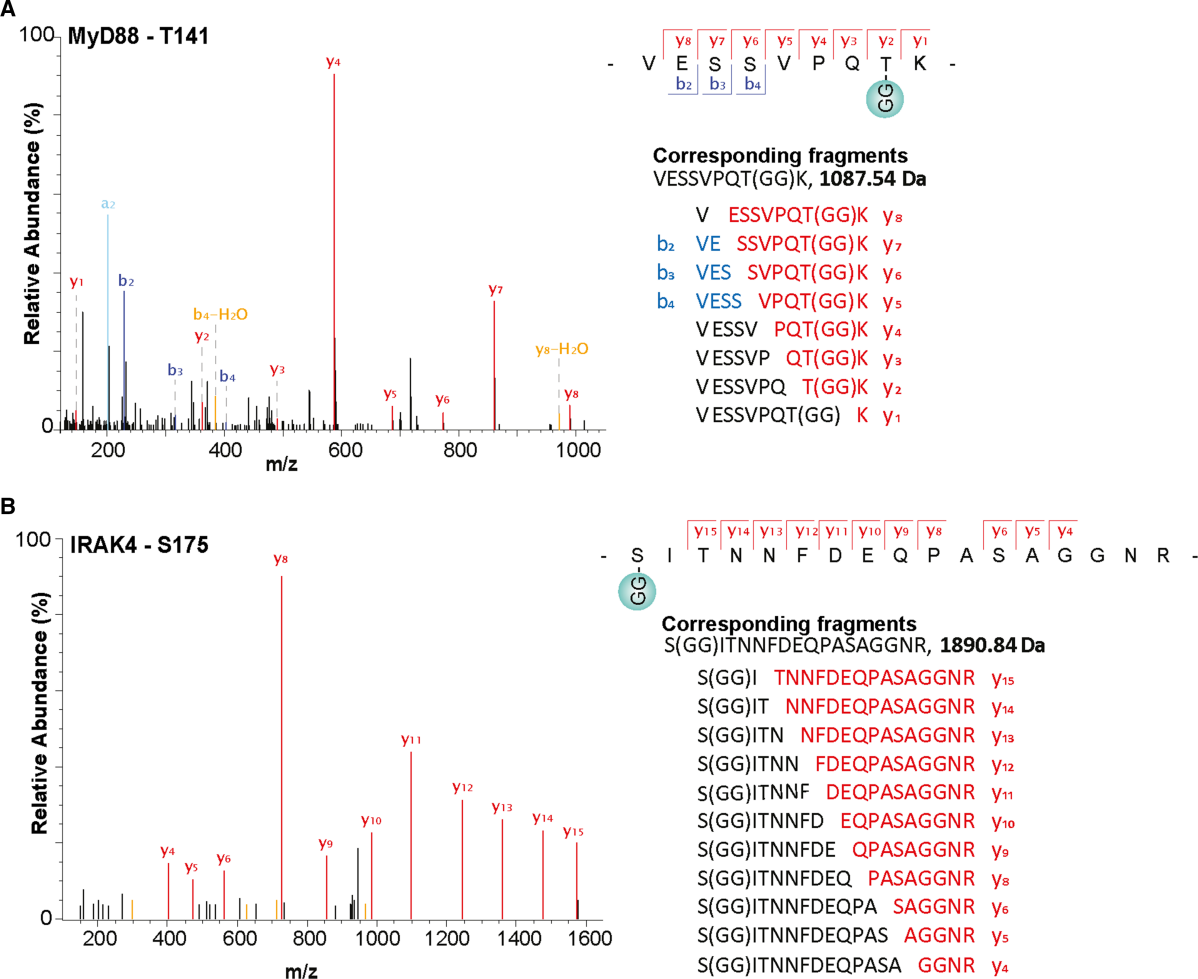
Serine and Threonine residues in MyD88 and IRAK4 that undergo R848-stimulated ubiquitylation in mouse RAW cells. (**A**,**B**) Mass spectromic fragmentation patterns of the indicated tryptic peptides from MyD88 (**A**) and IRAK4 (**B**). ‘GG' represents the remnant of Ubiquitin attached to the specified amino acid in the target protein generated after tryptic digestion. The identities of each of the fragment ions detected are shown to the right of the corresponding spectra. Data information: The spectra shown were obtained 20 min after stimulation with R848.

**Figure 3.**
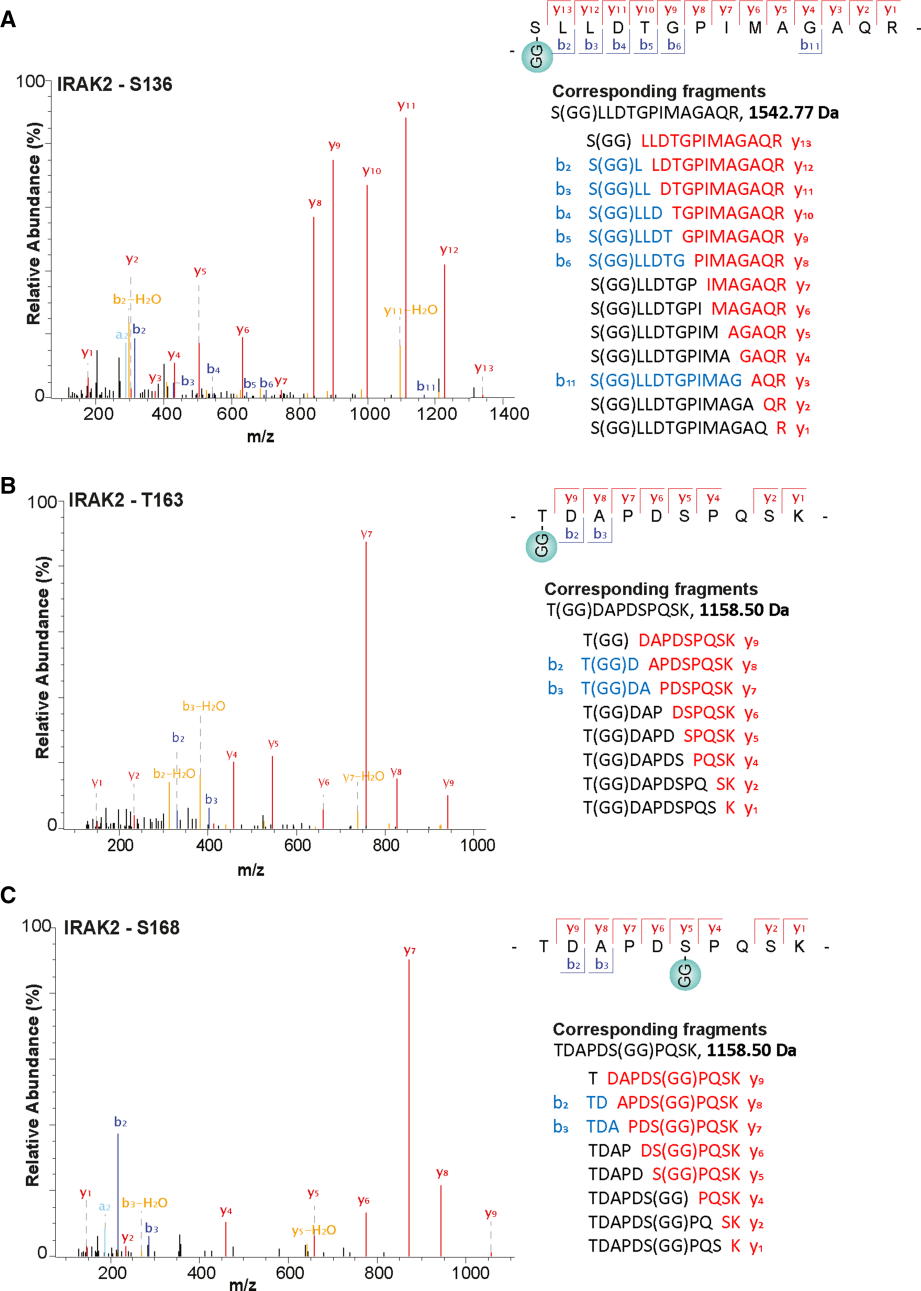
Serine and Threonine residues in IRAK2 that undergo R848-stimulated ubiquitylation in mouse RAW cells. (**A**–**C**) Mass spectromic fragmentation patterns of the indicated tryptic peptides from IRAK2. ‘GG' represents the remnant of Ubiquitin attached to the specified amino acid in the target protein generated after tryptic digestion. The identities of each of the fragment ions detected are shown to the right of the corresponding spectra. Data information: The spectra shown were obtained 20 min after stimulation with R848.

**Figure 4.**
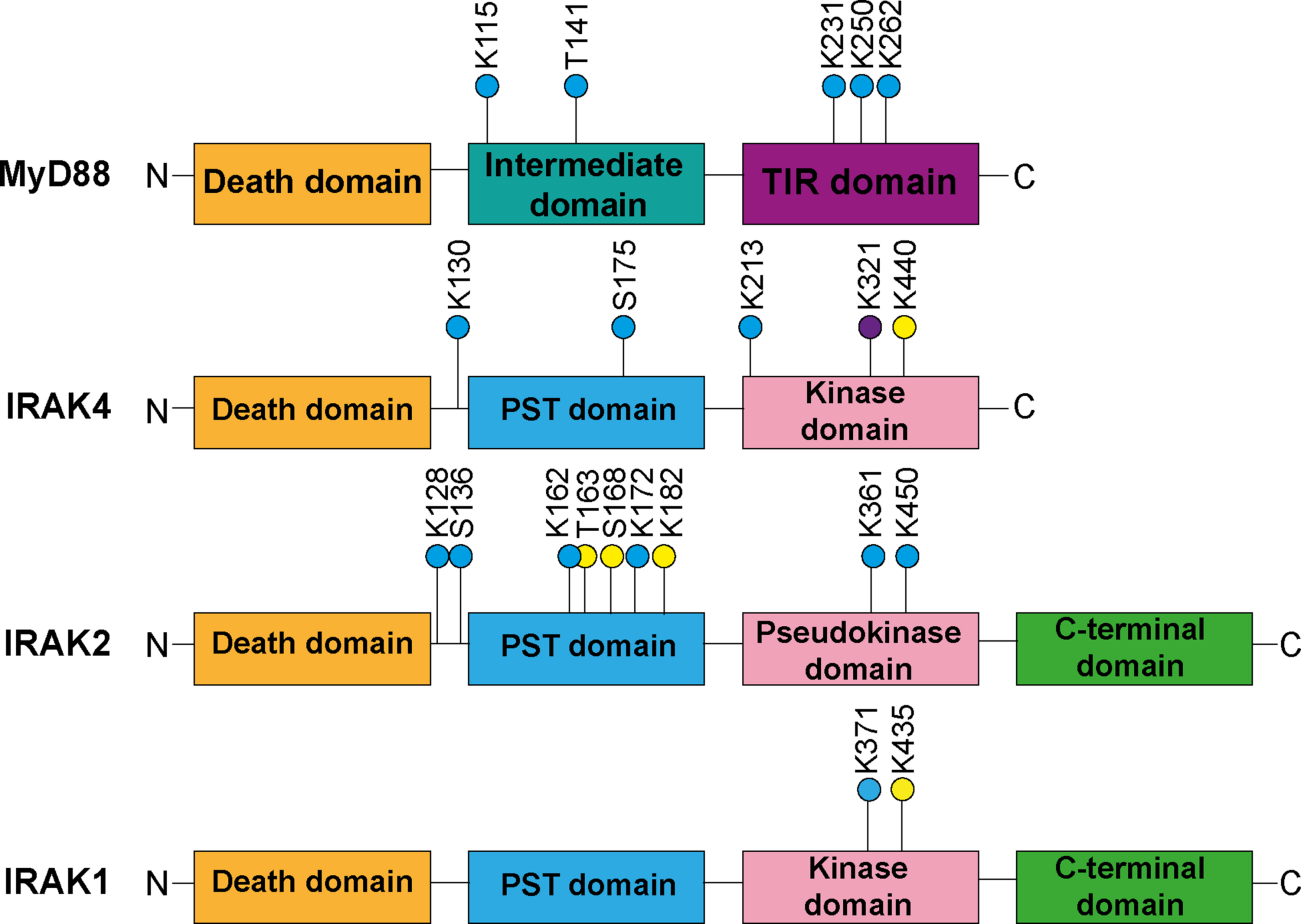
Location of ubiquitylated serine, threonine and lysine residues within the domain structures of IRAKs and MyD88. The sites of ubiquitylation identified after stimulation for 20 min only (yellow circles), 6 h only (purple circles) and identified at both 20 min and 6 h (blue circles) are shown. PST domain, proline, serine, threonine-rich domain; TIR domain, Toll Interleukin-1 receptor domain. The C-terminal domains of IRAK1 and IRAK2 contain TRAF6 interaction (Pro-Xaa-Glu) motifs where Xaa can be any amino acid.

The UbiSite method enabled us to identify sites of ester-linked ubiquitylation in MyD88, IRAK4 (Figure 2) and IRAK2 (Figure 3) and sites of isopeptide-linked ubiquitylation in MyD88 (Supplementary Figure S2) IRAK4 (Supplementary Figure S3), IRAK2 (Supplementary Figure S4) and IRAK1 (Supplementary Figure S5). The ubiquitylation sites were only detected in R848-stimulated cells and not in unstimulated cells. The sites of ester-linked ubiquitylation were confined to the proline, serine, threonine-rich (PST) region of IRAKs 4 and 2, and the intermediate domain of MyD88 (Figure 4). Lys162 of IRAK2 is adjacent to Thr163, and Thr163 is located within the same tryptic peptide as Ser168, but no diubiquitylated peptides were detected in which both Thr163 and Ser168, Lys162 and Thr163 or Lys162 and Ser168 were ubiquitylated; nor was a peptide detected in which all three sites were ubiquitylated. These observations suggest that the ubiquitylation of these sites is mutually exclusive and consequently that each of these three sites of ubiquitylation are attached to different IRAK2 molecules. Sites of isopeptide-linked ubiquitylation were not only found in the PST and intermediate domains, but also in the kinase domain of IRAK4 and IRAK1, the pseudokinase domain of IRAK2, and the TIR (Toll-interleukin receptor) domain of MyD88 (Figure 4). Together, these results establish that the ubiquitin chains attached to the components of myddosomes are indeed initiated by both ester and isopeptide bonds.

We did not detect any sites of ester-linked ubiquitylation in IRAK1, which should be present based on the sensitivity of polyubiquitylated IRAK1 to hydroxylamine (Figure 1A) [2]. The failure to identify ester bonds in IRAK1 may be explained if, like IRAK2 and IRAK4, they are located within the PST domain, because mouse IRAK1 only contains two lysine (Lys101 and Lys147) and two arginine residues (Arg100 and Arg160) in this region. Since ubiquitylation of a lysine residue prevents tryptic cleavage, the predicted peptide fragments derived from this region of IRAK1 would comprise amino acid residues 101–160 or 63–160, if Lys101 and Lys147 were both ubiquitylated and depending on whether the ubiquitylation of Lys101 prevents tryptic cleavage at Arg100. Peptides of this size can be difficult to detect by MS and suggests that digestion with an additional protease will be needed to generate smaller ubiquitylated peptides that can be identified. Lys134 and Lys180 of human IRAK1, the residues equivalent to Lys101 and Lys147 of mouse IRAK1, are reported to be major and minor sites of isopeptide ubiquitylation, based on overexpression studies in HEK293 cells in which either or both of these lysine residues were mutated to arginine [32].

### Ester linkages are formed between two ubiquitin molecules

**Figure 5.**
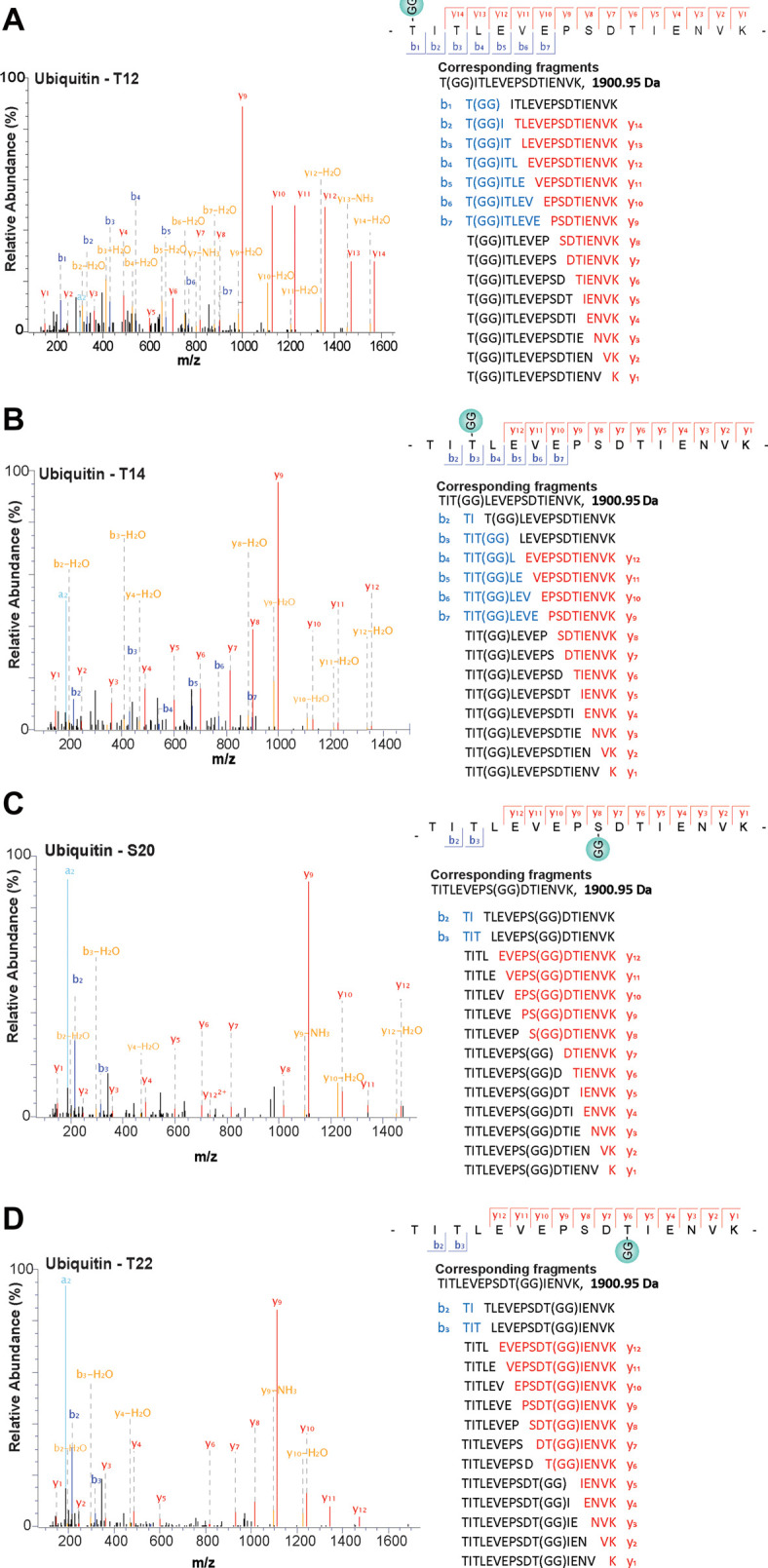
Ubiquitin modified by ester linkages are formed in R848-stimulated RAW cells. (**A**–**D**) Mass spectromic fragmentation patterns of peptides comprising amino acid residues 12–27 of ubiquitin, each containing a ‘GG' signature. Their fragmentation patterns show that they are ubiquitylated at Thr12, Thr14, Ser20 or Thr22. The identities of each of the fragment ions detected are shown to the right of the corresponding spectra. Data information: The spectra shown were obtained 20 min after stimulation with R848.

**Figure 6.**
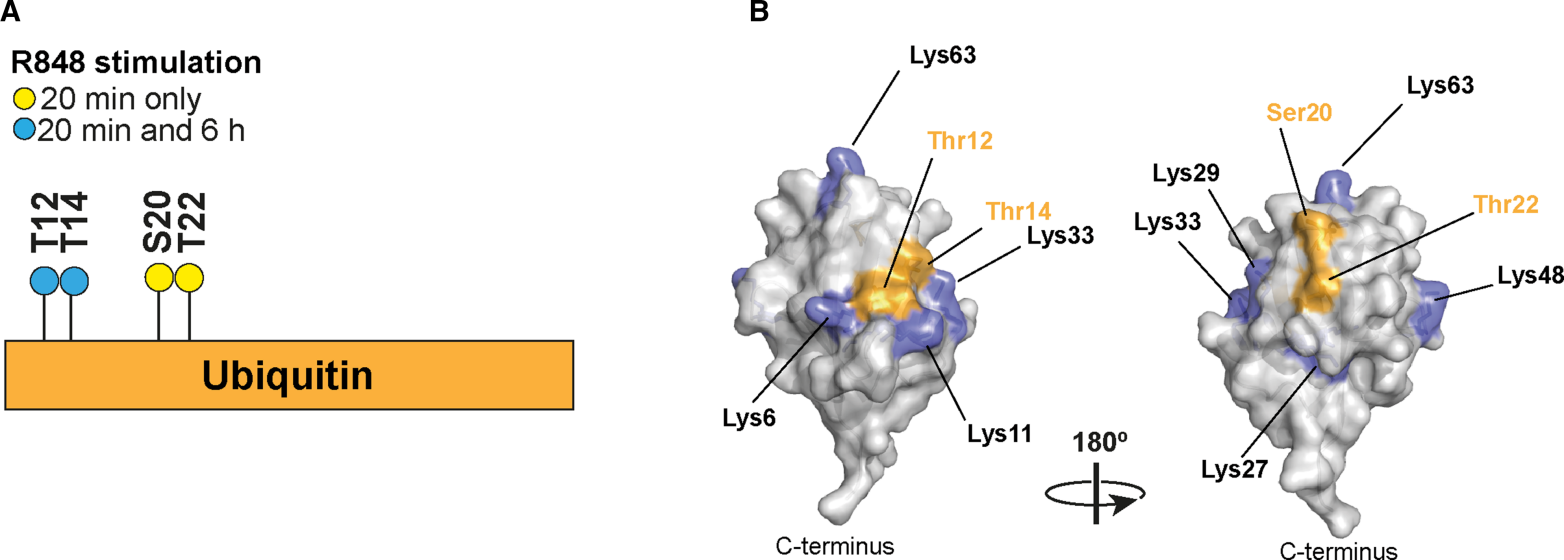
The four inter-ubiquitin ester bonds formed during TLR7 signalling and their location on the surface of ubiquitin. (**A**) Schematic showing the time at which each modified ubiquitin was identified after stimulation with R848. All four ester-linked ubiquitylation sites were detected after stimulation for 20 min with R848 (yellow circles) but Thr12 and Thr14 were the only ubiquitylation sites detected after stimulation for 6 h (blue circles). The ubiquitylation of Thr14 was also detected in unstimulated cells. (**B**) The three-dimensional structure of ubiquitin (PDB: 1UBQ) showing the location of Thr12, Thr14, Ser20 and Thr22 and the seven lysine residues [4].

The only other protein that we detected in the Halo-NEMO pull downs that was linked to ubiquitin by an ester bond was ubiquitin itself. Remarkably, we found that four different Ser/Thr residues in ubiquitin formed ester bonds with the C-terminus of another ubiquitin molecule namely Thr12 (Figure 5A), Thr14 (Figure 5B), Ser20 (Figure 5C) and Thr22 (Figure 5D). The linkages between Thr12, Ser20 and Thr22 were detected after stimulation for 20 min with R848 and the R848-dependent ubiquitylation of Thr12 was also detected after stimulation for 6 h (Figure 6A). The ubiquitylation of Thr14 was also identified in unstimulated cells.

Until now, eight types of ubiquitin linkage have been identified in cells in which the C-terminus of one ubiquitin is linked to the ε-amino groups of any of the seven lysine residues or to the α-amino group of the N-terminal methionine of another ubiquitin. The present study, therefore, increases the number of ubiquitin linkage types that are formed in cells from eight to 12. HOIL-1 is probably the E3 ligase that ubiquitylates Thr12, Ser20 and Thr22 because it has been shown to form these three ubiquitin linkage types *in vitro* [2]. Whether HOIL-1 is also the E3 ligase that ubiquitylates Thr14 of ubiquitin will require further experimentation. The LUBAC-catalysed formation of an ester bond between Thr55 of ubiquitin and the C-terminal carboxylate of another ubiquitin molecule *in vitro* has also been reported [3]. We did not detect ester bonds linking Thr55 of ubiquitin to another ubiquitin molecule in our experiments, but this does not exclude the possibility that this linkage type is formed in response to other stimuli and/or in other cells. Although Thr12, Thr14, Ser 20 and Thr22 are all located in the same tryptic peptide comprising amino acid residues 12–27 of ubiquitin, we did not detect any peptide that was ubiquitylated at more than one site. Thus, four distinct ubiquitin linkage types produced by ester bonds are formed when BMDM are stimulated with R848.

It is a paradigm that different ubiquitin linkage types adopt distinct conformations that are recognised by specific ubiquitin-binding proteins, and which function to decode the ubiquitin signal (reviewed [33]). Thr12/Thr14 and Ser20/Thr22 are situated on the surface of ubiquitin but on opposite sides of the molecule (Figure 6B). This suggests that the structures of these two pairs of ubiquitin linkages are likely to differ markedly and, consequently, may interact with different decoding proteins. It will clearly be important to determine the structures of each ester-linked ubiquitin dimer by X-ray crystallography and to identify their binding partners in cells. The binding partners may include proteins that stimulate or suppress innate immune signalling and explain why HOIL-1-deficient humans display a combination of immunodeficiency and auto-inflammation [17,18].

Interestingly, ubiquitin modified via Thr12, Ser20 and Thr22 were detected after stimulation with R848 for 20 min, but only Thr12 was detected after 6 h (Figure 6A). It is tempting to speculate that this difference may be related to the distinct functions of these linkage types. For example, we have suggested that the initial phase of TLR signalling up to 2 h not only initiates inflammatory mediator production but also functions to activate, recruit and induce negative regulators of innate immunity. Such molecules include the IKK-related kinases (TBK1 and IKKε) [34,35], the ubiquitin-binding proteins A20 and ABIN1 (reviewed in [36]) and the dual specificity phosphatase 1 (DUSP1) [37]. These negative regulators prevent the overproduction of inflammatory mediators during the late phase of signalling (2–8 h) [38], which would otherwise lead to inflammatory and autoimmune diseases. It is, therefore, tempting to speculate that ubiquitin molecules linked via Ser20 or Thr22 might recruit negative regulators of TLR signalling, while ubiquitin linked by Thr12 may recruit proteins that enhance inflammatory mediator production. The failure to detect ubiquitin molecules linked via Ser20 and Thr22 after 6 h of TLR stimulation also suggests that these ubiquitin linkage types may be hydrolysed more rapidly than ubiquitin molecules linked via Thr12 and/or by different DUBs.

The identities of the DUB(s) that hydrolyse the ester-linked ubiquitin bonds that we have detected is another interesting topic for future research. Several DUBs of the USP (ubiquitin-specific protease) family, the Machado–Joseph disease (MJD) family, and vOTU, a virally encoded member of the OTU (ovarian tumour) family of DUBs, have been shown to hydrolyse a ubiquitylated threonine substrate linked by an ester bond *in vitro* [39]. However, USP family members and vOTU also hydrolyse isopeptide bonds, indicating that ester and isopeptide-linked ubiquitin may not necessarily be deubiquitylated by different DUBs.

The ubiquitin linkages formed by ester bonds were identified by UbiSite analysis after first capturing them on Halo-NEMO resin. Since NEMO is thought to interact with M1-Ub linkages specifically, this suggests that ubiquitin linkages produced by the formation of ester bonds may be attached covalently to M1-Ub oligomers and/or to K63/M1-Ub hybrids that are also captured by Halo-NEMO resin [24]. The high molecular mass ubiquitin chains formed when mouse embryonic fibroblasts are stimulated with tumour necrosis factor (TNF) and detected by immunoblotting with an M1-Ub-specific antibody, also contain ester linkages as judged by the reduction in their size after hydroxylamine treatment [3]. However, whether the ester-linked ubiquitin is attached to M1-Ub and/or K63-Ub linkages, to each other, or to other proteins not detected in our study, will require further analysis of the composition and topology of the ubiquitin chains formed in TLR-stimulated cells.

Interestingly, Ser136 of IRAK2, one of the sites of ubiquitylation identified in this study, has been reported to undergo phosphorylation when mouse BMDM are stimulated with lipopolysaccharide, an activator of TLR4 [40]. Moreover, the phosphorylation of Thr12 [41], Thr14 [42], Ser20 [43] and Thr22 [44] of ubiquitin have all been reported in a variety of mammalian cells. Clearly, one potential role for the phosphorylation of these sites could be to prevent their ubiquitylation but, conversely, ubiquitylation can prevent phosphorylation. Since ubiquitylation and phosphorylation of the same amino acid residue is mutually exclusive, this could be an interesting way in which protein kinases regulate the actions of E3 ligases forming ester bonds and vice versa.

In summary, the present study is, to our knowledge, the first to exploit UbiSite technology to identify specific amino acid residues in proteins that undergo ubiquitylation in response to an extracellular signal and demonstrates its utility for this type of analysis.

## Methods

### Plasmids and proteins

The plasmids utilised in this study and glutathione-S-transferase (GST) fusions of human GST-Otulin (DU43487), human AMSH-LP[264–436] (DU15780) and GST-USP2 (DU13025) were produced by MRC Reagents and Services and can be requested at http://mrcppureagents@dundee.ac.uk. The preparation of Halo-tagged NEMO and its covalent attachment to Halo-Link Resin (Promega) have been described [24]. LysC proteinase was from Thermo Fischer Scientific (#90051), trypsin from Promega (#V511A) and the protein phosphatase from bacteriophage λgt10 (phage λ phosphatase) [45] from New England Biolabs (#P0753S).

### Antibodies

The antibodies recognising IRAK1 (Cell Signalling Technology #4504S) and IRAK2 (Abcam #ab62419) were purchased from the sources indicated in parentheses. The antibody recognising IRAK4 phosphorylated at both Thr345 and Ser346 was a gift from Dr Vikram Rao (Pfizer, Boston, MA, U.S.A.). This antibody was used in this study because it is more sensitive than any commercially available IRAK4 antibody that we have tested.

### Cell culture, stimulation and lysis

RAW264.7 cells (RAW cells) were maintained in Dulbecco's modified Eagle's medium (DMEM) supplemented with 10% (v/v) foetal bovine serum, 2 mM l-glutamine, 100 Units/ml penicillin and 0.1 mg/ml streptomycin. Bone marrow from HOIL-1[C458S] mice [2] and wild-type littermates was differentiated into primary BMDM using L929 preconditioned medium [38]. Cells were stimulated with or without 250 ng/ml R848, washed rapidly with ice-cold phosphate-buffered-saline and lysed in 50 mM Tris–HCl pH 7.5, 1 mM EGTA, 1 mM EDTA, 1% (v/v) Triton X-100, 270 mM sucrose, containing the protein phosphatase inhibitors 10 mM glycerol-2-phosphate, 50 mM sodium fluoride, 5 mM sodium pyrophosphate, 1 mM sodium orthovanadate and the proteinase inhibitors 1 mM phenylmethanesulphonyl fluoride, cOmplete protease inhibitor (one tablet per 50 ml) (Roche) and 100 mM iodoacetamide to inhibit DUB activities. Cell lysates were centrifuged at 17 000×***g*** for 15 min at 4°C and the supernatant, termed cell extract was removed and used for experimentation. Protein concentrations in cell extracts were measured by the Bradford Assay [46].

### Capture of proteins on Halo-NEMO beads and treatment with deubiquitylases and hydroxylamine

Halo-NEMO resin [24] was washed twice with 6 M urea solution and five times with 50 mM Tris–HCl pH 7.5, 1% (v/v) Triton X-100. Following incubation overnight with cell extract (2 mg per 20 µl packed Halo-NEMO resin), the resin was collected by centrifugation, washed twice with 50 mM Tris–HCl pH 7.5, 500 mM NaCl, 1% (v/v) Triton X-100 and twice with 50 mM Tris–HCl pH 7.5, 1% (v/v) Triton X-100 and the supernatant discarded.

The packed beads were resuspended in 50 mM HEPES pH 7.5, 100 mM NaCl, 2 mM dithiothreitol, 0.01% (w/v) Brij-35 and incubated for 1 h at 37°C without or with one or more of the DUBs Otulin, AMSH-LP and USP2 (each at 1 µM) as indicated in the figure legends. The beads were washed twice with 50 mM Tris–HCl pH 7.5, 1% (v/v) Triton X-100, once with 50 mM Tris–HCl pH 7.5 and resuspended in 19 mM sodium carbonate, 22 mM sodium bicarbonate pH 9.0 and incubated for 1 h at 37°C with or without 0.5 M hydroxylamine. The beads were washed twice with 50 mM Tris–HCl pH 7.5, 1% (v/v) Triton X-100 and proteins eluted by resuspension for 10 min at 37°C in Lithium Dodecyl Sulfate sample buffer (Invitrogen) diluted four-fold in 2.5% (v/v) 2-mercaptoethanol. The eluted proteins were passed through a Spin-X centrifuge filter (Corning Costar) and subjected to SDS–PAGE using precast 4–12% gels and transferred to PVDF membranes prior to immunoblotting.

### Mapping of ubiquitylated amino acid residues using the UbiSite antibody

Two-hundred and fifty milligrams cell extract protein was incubated with 2 ml packed Halo-NEMO beads. After completion of the washing steps, the beads were resuspended in 50 mM HEPES pH 7.5, 100 mM NaCl, 2 mM DTT, 0.01% (w/v) Brij-35 and incubated for 30 min at 37°C with 1000 U of phage λ phosphatase. The beads were washed twice with 50 mM Tris–HCl pH 7.5, 1% (v/v) Triton X-100, five times with 50 mM Tris–HCl pH 7.5, and then incubated for 5 min at 37 °C with 5% (w/v) Rapigest. After diluting five-fold with 50 mM Tris–HCl pH 7.5 and incubation for 10 min at 37°C, the eluted proteins were separated from the beads using a Spin-X centrifuge filter. The flowthrough containing the eluted proteins was made 2 mM in DTT, incubated for 30 min at room temperature and then alkylated for a further 30 min with 11 mM chloroacetamide (CAA). The samples were diluted 10-fold with 50 mM Tris–HCl pH 7.5 to reduce the Rapigest concentration to 0.1%, then digested overnight at 37°C with LysC proteinase at a concentration of 1 : 100 (w/w) enzyme to protein. Digestion was terminated by the addition of trifluoroacetic acid to a final concentration of 2% (v/v) and incubated for 1 h at 37°C. Following centrifugation for 10 min at 10 000×***g***, the supernatant was removed and desalted using a WATERS C_18_ Cartridge (WAT054955) according to the manufacturer's instructions. The eluted peptides were lyophilised, reconstituted in 50 mM MOPS pH 7.2, 10 mM Na_2_HPO_4_, 50 mM NaCl, enriched by immunoprecipitation with the UbiSite antibody and analysed as described before [25].

Mass spectrometric analyses were performed essentially as described [47], with a few modifications. An Orbitrap Exploris 480 mass spectrometer (ThermoFisher Scientific) was used in a data-dependent mode by shifting from full-scan event to the top 12 MS/MS scans. The instrument was operated in positive polarity mode with the following parameters for full-scan acquisitions: the normalised automatic gain control (AGC) target value was set to 300%, resolution was 60 000 with a scan range of 350–1500 m/z and a maximum ion injection time of 25 ms. The ion charge range was set from 2 to 6 charges. MS/MS fragmentation of precursors was obtained by HCD (higher-energy collisional dissociation) with a normalised collisional energy of 30. Resolution of MS/MS scans was 30 000, maximum IT of 54 ms and the isolation window was 1.2 m/z. The dynamic exclusion window was set to 60 se to prevent the same peptide sequences from repeating. Raw MS files were processed through Proteome Discoverer software (P.D. version 2.5.0.400; ThermoFisher Scientific) using the Sequest HT search engine with the following parameters: the precursor mass tolerance was set to 10 ppm and the fragment mass tolerance was 0.02 Da, the protease trypsin was allowed a maximum of two missed cleavage sites and a minimum peptide length of six amino acid residues. Carbamidomethylation (+57.021 Da) of Cysteine residues was set as fixed modification. Oxidation of Methionine (+15.995 Da) and Gly-Gly modification on Lysine, Serine and Threonine (+114.043 Da) were set as variable modifications. Data were searched against the Uniprot Mouse database (from April 2022) using the Percolator node of P.D. software to estimate FDR (less than 1%). All spectra with diGly modifications on serine and threonine residues presented in the manuscript were verified manually.

## Data Availability

All data, including annotated spectra for all mass spectrometric identifications, has been provided in the main article and in Supplementary Material.

## Open Access

Open access for this article was enabled by the participation of University of Dundee in an all-inclusive *Read & Publish* agreement with Portland Press and the Biochemical Society under a transformative agreement with JISC.

## Abbreviations

ABIN: A20-binding inhibitor of NF-κB
AMSH-LP: associated molecule with the SH3-domain of STAM-like protease
BMDM: bone marrow-derived macrophages
DUB: deubiquitylase
GST: glutathione S-transferase
HOIL-1: haem-oxidised IRP2 ubiquitin ligase-1
HOIP: HOIL-1-interacting protein
IKK: IκB kinase
IL: interleukin
IRAK: interleukin receptor-associated kinase
IRF5: interferon regulatory factor 5
ISG15: interferon-stimulated gene 15
K63-Ub: ubiquitin linked via lysine 63
LUBAC: linear ubiquitin assembly complex
M1-Ub: ubiquitin linked via methionine 1
MJD: Machado-Joseph disease
MS: mass spectrometry
MyD88: myeloid differentiation primary response 88
NEDD8: neuronal precursor cell-expressed developmentally down-regulated protein 8
NEMO: NF-κB essential modulator
OTU: ovarian tumour
PST: proline serine threonine
RBCK1: RING-B-box-coiled-coil protein interacting with PKC 1
TAK1: TGFβ-activated kinase
TIR: Toll-interleukin receptor
TLR: Toll-like receptor
TRAF6: TNF receptor associated factor 6
UBL: ubiquitin-like
USP: ubiquitin-specific protease
