# Supplementary material for: Identification of ester-linked ubiquitylation sites during TLR7 signalling increases the number of inter-ubiquitin linkages from 8 to 12
Source: Biochem J. 2022 Dec 7;479(23):2419–31. doi: 10.1042/BCJ20220510 (PMC9788571; doi:10.1042/BCJ20220510)
Supplement: Supplementary Material [file BCJ-479-2419-s1.pdf]

Supplementary Figure Legends

Fig S1. Work-flow describing the methodology used to identify sites of endogenous Ser/Thr and Lys ubiquitylation in cells in a single experiment.

Fig S2. Isopeptide-linked ubiquitylation sites in MyD88 formed in response to R848 in mouse RAW cells.
Mass fragmentation patterns of the indicated tryptic peptides from MyD88. “GG” represents the remnant of Ubiquitin attached to the specified amino acid in the target protein generated after trypsin digestion. Data information: the spectra shown were obtained 20 min after stimulation with R848.

Fig S3. Isopeptide-linked ubiquitylation sites in IRAK4 formed in response to R848 in mouse RAW cells.
Mass fragmentation patterns of the indicated tryptic peptides from IRAK4. “GG” represents the remnant of Ubiquitin attached to the specified amino acid in the target protein generated after trypsin digestion. Data information: The spectra shown were obtained 20 min after stimulation with R848 except for the peptide containing Lys321 which was obtained after stimulation for 6 h.

Fig S4. Isopeptide-linked ubiquitylation sites in IRAK2 formed in response to R848 in mouse RAW cells.
Mass fragmentation patterns of the indicated tryptic peptides from IRAK2. “GG” represents the remnant of Ubiquitin attached to the specified amino acid in the target protein generated after trypsin digestion. Data information: The spectra shown were obtained 20 min after stimulation with R848.

Fig S5. Isopeptide-linked ubiquitylation sites in IRAK1 formed in response to R848 in mouse RAW cells.
Mass fragmentation patterns of the indicated tryptic peptides from IRAK1. “GG” represents the remnant of Ubiquitin attached to the specified amino acid in the target protein generated after trypsin digestion. Data information: The spectra shown were obtained 20 min after stimulation with R848

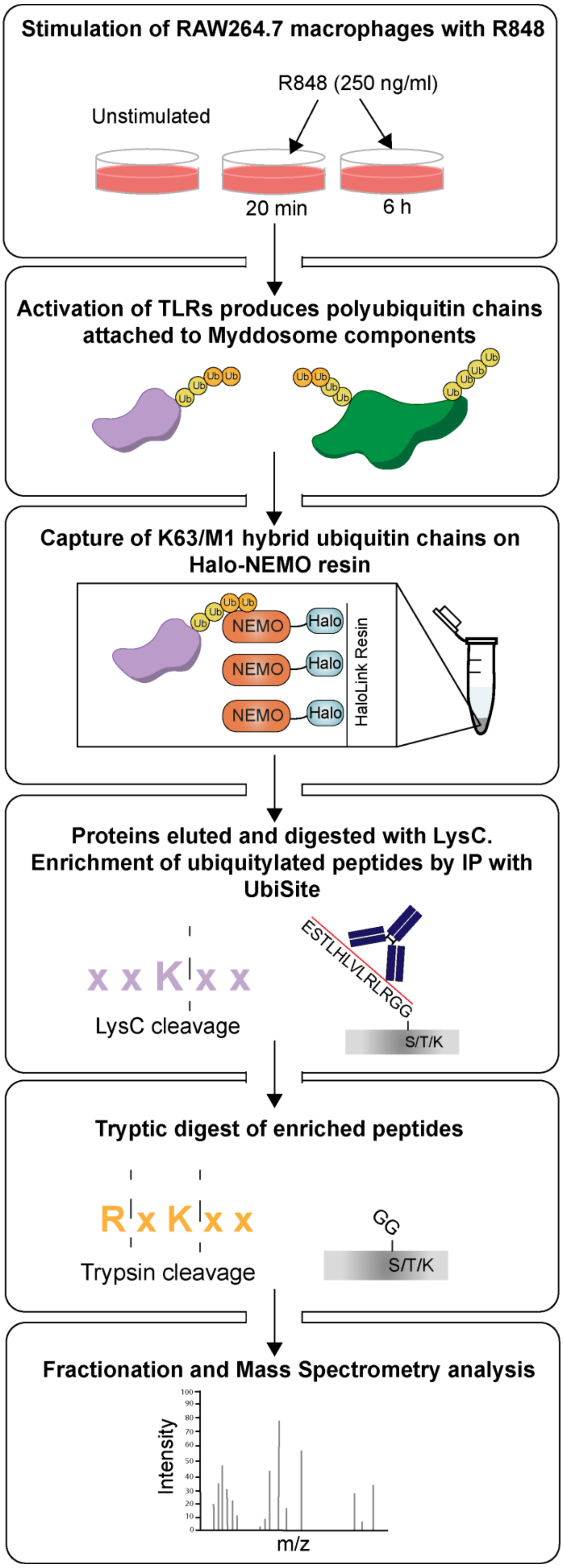
Stimulation of RAW264.7 macrophages with R848
R848 (250 ng/ml)
Unstimulated
20 min
6 h
Activation of TLRs produces polyubiquitin chains attached to Myddosome components
Ub
Capture of K63/M1 hybrid ubiquitin chains on Halo-NEMO resin
NEMO
Halo
HaloLink Resin
Proteins eluted and digested with LysC. Enrichment of ubiquitylated peptides by IP with UbiSite
x x K x x
LysC cleavage
ESTLHLVLRLRGG
S/T/K
Tryptic digest of enriched peptides
R x K x x
Trypsin cleavage
GG
S/T/K
Fractionation and Mass Spectrometry analysis
Intensity
m/z

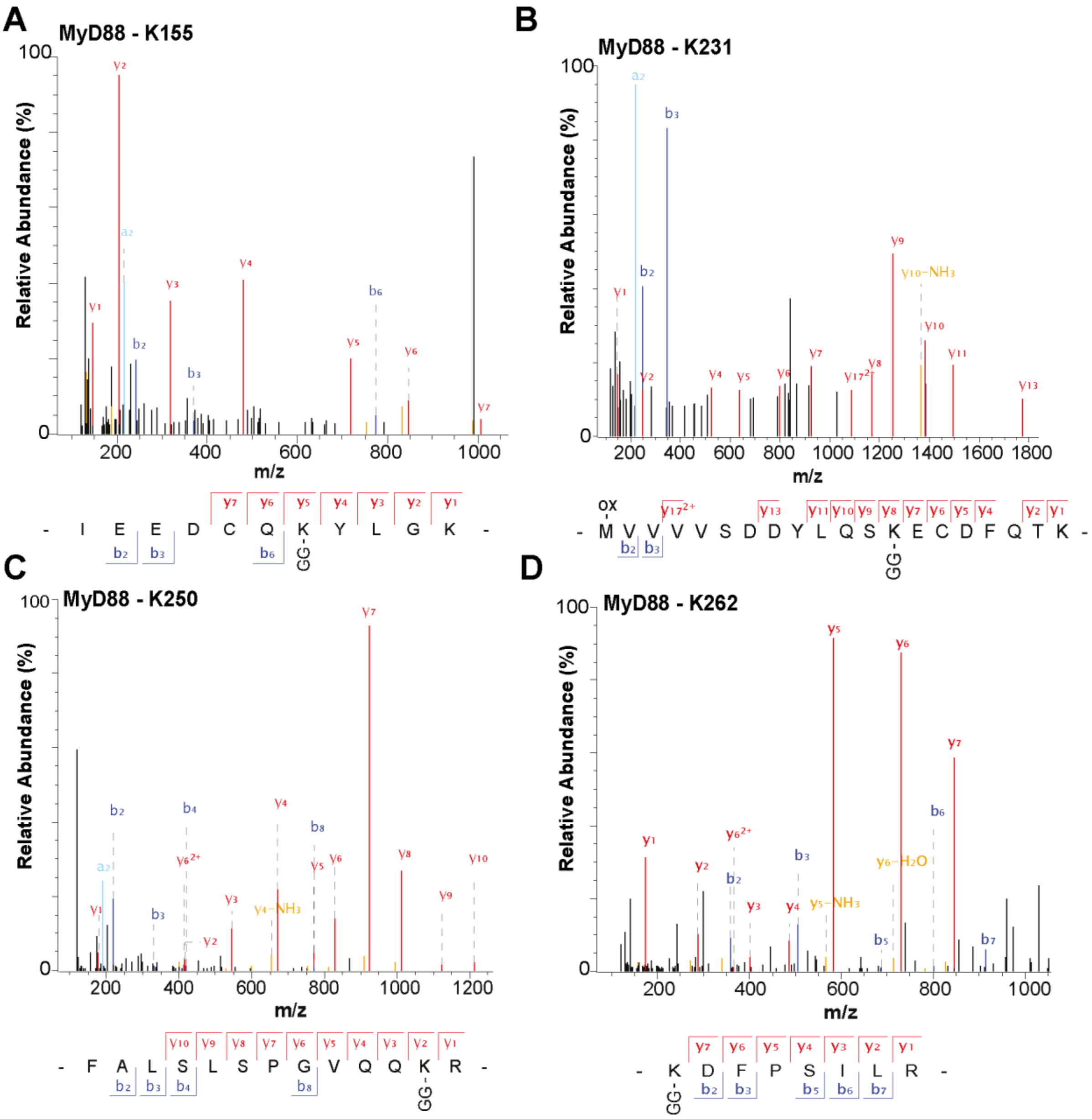
A
MyD88 - K155
Relative Abundance (%)
m/z
- I E E D C Q K Y L G K -
GG-
B
MyD88 - K231
Relative Abundance (%)
m/z
ox
- M V V V V S D D Y L Q S K E C D F Q T K -
GG-
C
MyD88 - K250
Relative Abundance (%)
m/z
- F A L S L S P G V Q Q K R -
GG-
D
MyD88 - K262
Relative Abundance (%)
m/z
- K D F P S I L R -
GG-

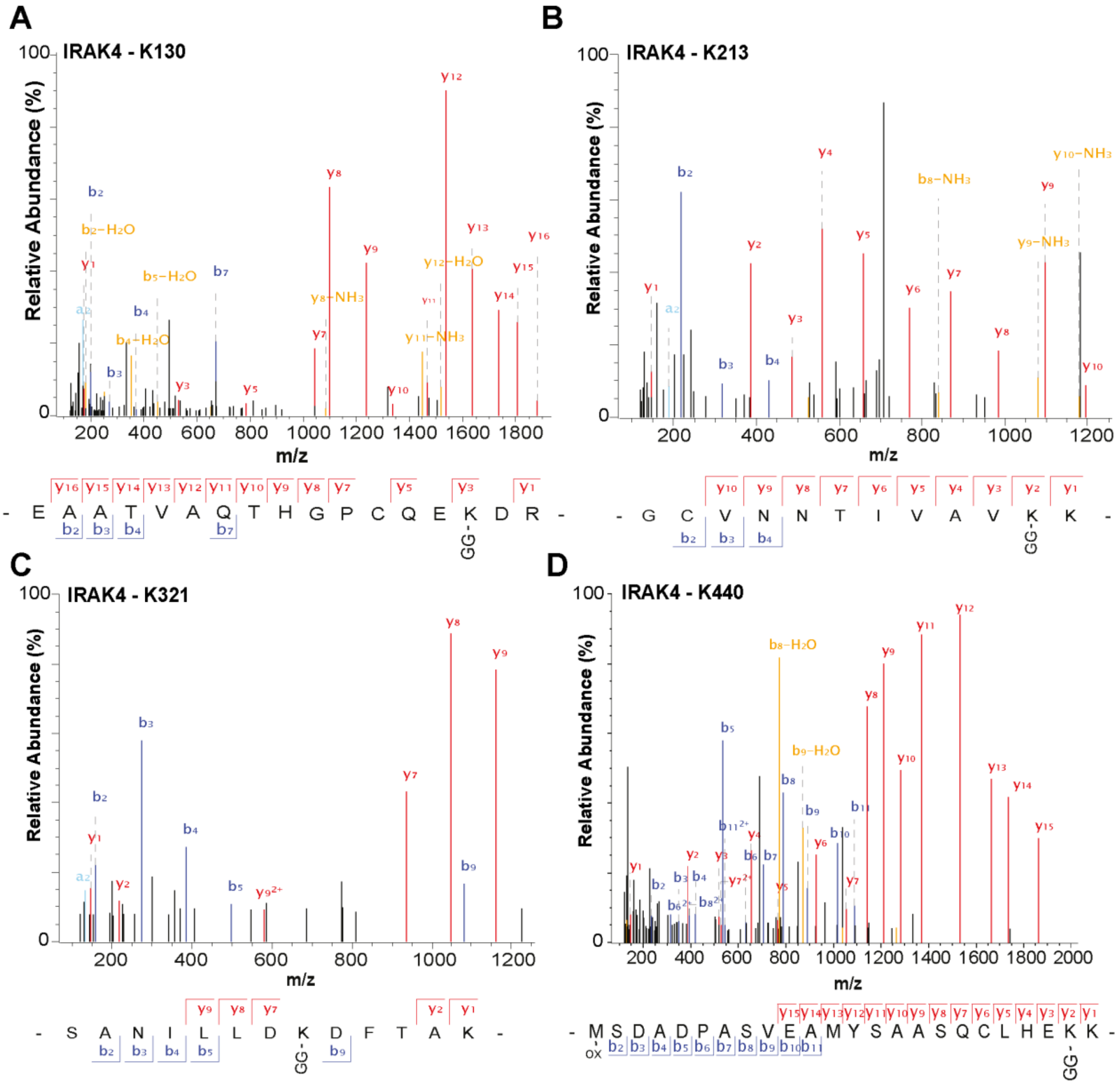
A
IRAK4 - K130
Relative Abundance (%)
m/z
- E A A T V A Q T H G P C Q E K D R -
GG-
B
IRAK4 - K213
Relative Abundance (%)
m/z
- G C V N N T I V A V K K -
GG-
C
IRAK4 - K321
Relative Abundance (%)
m/z
- S A N I L L D K D F T A K -
GG-
D
IRAK4 - K440
Relative Abundance (%)
m/z
- M S D A D P A S V E A M Y S A A S Q C L H E K K -
ox
GG-

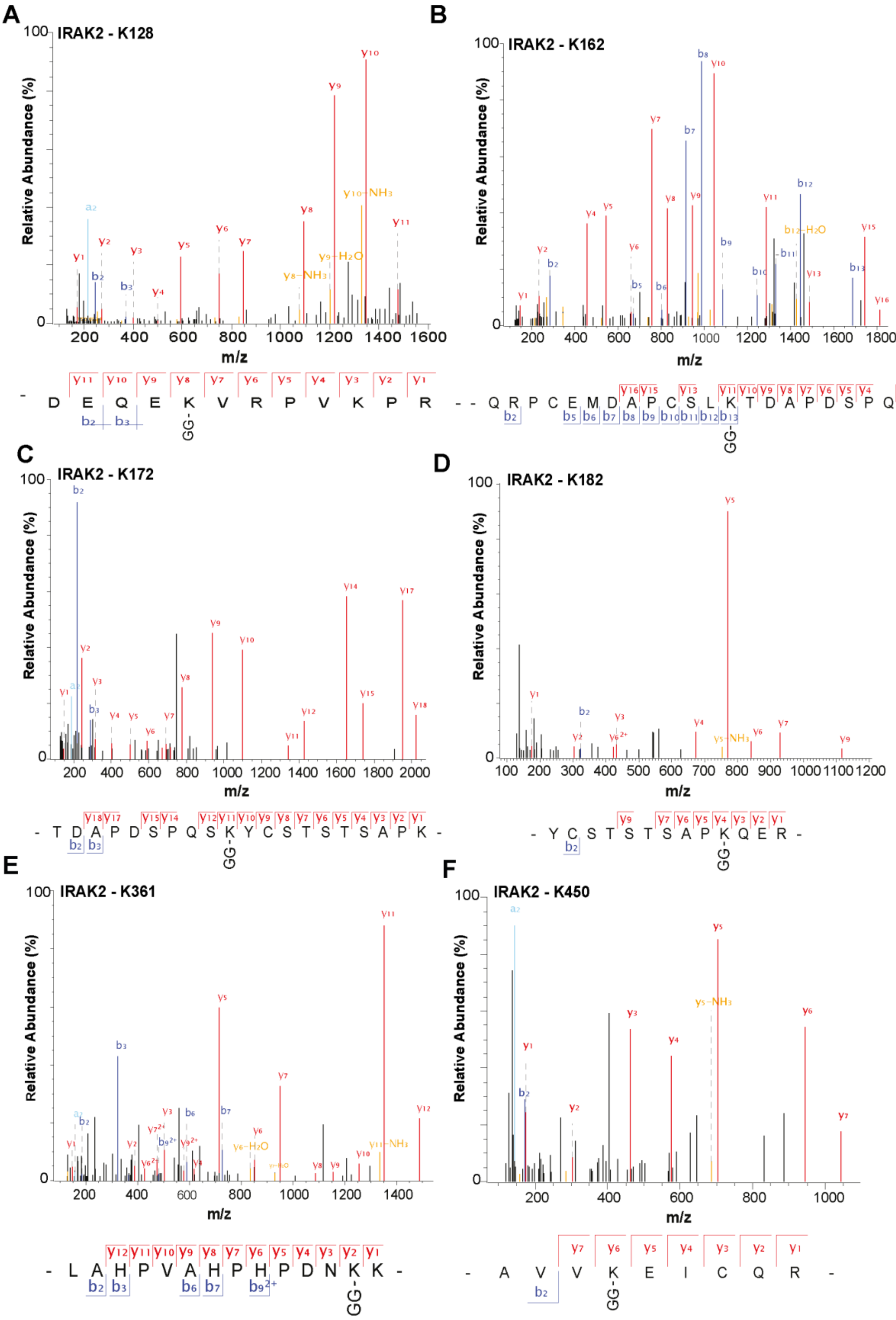
A
IRAK2 - K128
Relative Abundance (%)
m/z
- D E Q E K V R P V K P R
GG-
B
IRAK2 - K162
Relative Abundance (%)
m/z
- - Q R P C E M D A P C S L K T D A P D S P Q S K -
GG-
C
IRAK2 - K172
Relative Abundance (%)
m/z
- T D A P D S P Q S K Y C S T S T S A P K -
GG-
D
IRAK2 - K182
Relative Abundance (%)
m/z
- Y C S T S T S A P K Q E R -
GG-
E
IRAK2 - K361
Relative Abundance (%)
m/z
- L A H P V A H P H P D N K K -
GG-
F
IRAK2 - K450
Relative Abundance (%)
m/z
- A V V K E I C Q R -
GG-

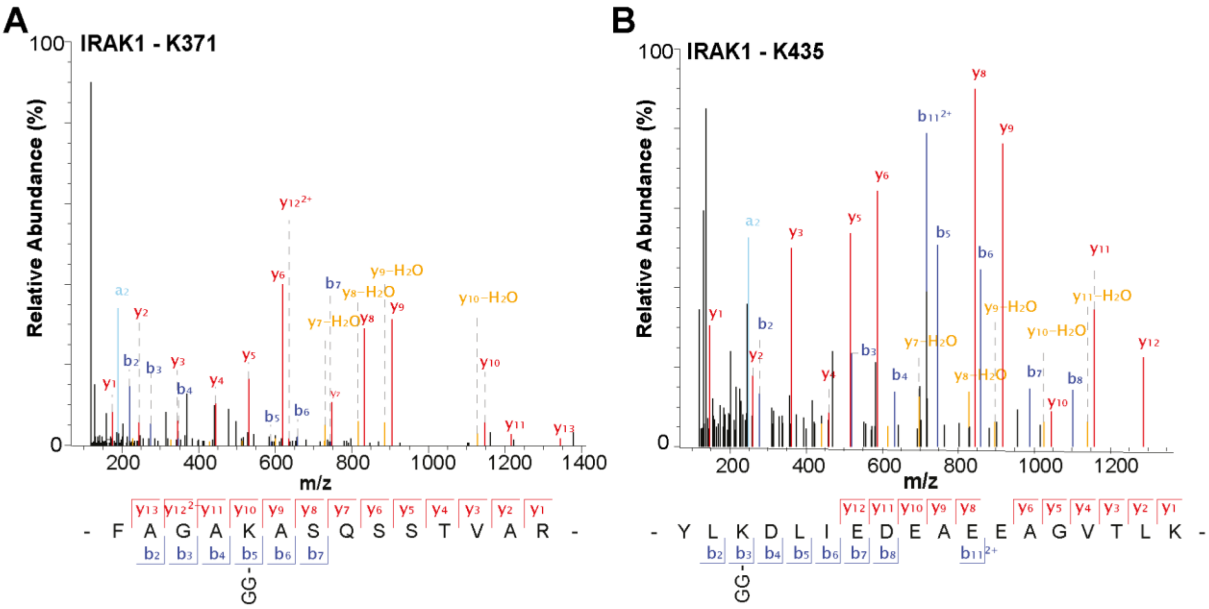
A
IRAK1 - K371
Relative Abundance (%)
m/z
- F A G A K A S Q S S T V A R -
GG-
B
IRAK1 - K435
Relative Abundance (%)
m/z
- Y L K D L I E D E A E E A G V T L K -
GG-
